# 数字引流系统在肺切除术后胸管管理中的应用现状与展望

**DOI:** 10.3779/j.issn.1009-3419.2026.101.16

**Published:** 2026-07-20

**Authors:** Yizhe WANG, Hongru SHEN, Jing KANG, Mohan SUN, Yong CUI, Peihao WANG

**Affiliations:** ^1^100050 北京，首都医科大学附属北京友谊医院胸外科; ^1^Department of Thoracic Surgery, Beijing Friendship Hospital, Capital Medical University, Beijing 100050, China; ^2^710038 西安，空军军医大学唐都医院胸腔外科; ^2^Department of Thoracic Surgery, Tangdu Hospital, Air Force Military Medical University, Xi'an 710038, China

**Keywords:** 肺肿瘤, 胸腔引流, 数字引流系统, 肺切除术, 加速康复外科, Lung neoplasms, Chest drainage, Digital drainage system, Pulmonary resection, Enhanced recovery after surgery

## Abstract

胸腔引流管理是肺切除术后围手术期管理的重要环节，旨在排除胸膜腔内积气及积液、促进肺复张。然而，传统胸腔引流系统主要依赖人工观察，难以连续量化漏气程度，导致胸管拔除时机判断存在主观性。近年来，数字引流系统（digital drainage system, DDS）逐渐应用于临床，其通过传感器连续监测胸腔压力及气、液流量，并生成动态趋势曲线，为胸腔引流管管理提供客观依据。DDS有助于提高引流评估的精准性，优化胸管管理策略，但其推广仍受到成本、应用经验及医疗体系差异等因素影响。本文对DDS在肺切除术后胸腔引流管理中的应用现状、优势及挑战进行综述。

## Abstract (en)

Chest drainage management is an essential component of perioperative care after pulmonary resection, aiming to evacuate intrapleural air and fluid and promote lung re-expansion. However, conventional chest drainage systems mainly rely on manual observation, making continuous and quantitative assessment of air leakage difficult and resulting in subjective decision-making regarding the timing of chest tube removal. In recent years, digital drainage systems (DDS) have been increasingly applied in clinical practice. By using integrated sensors, DDS enables continuous monitoring of pleural pressure, air flow, and fluid drainage, and provides dynamic trend curves, thereby offering objective evidence for chest tube management. DDS may improve the accuracy of drainage assessment and optimize chest tube management strategies; however, its widespread adoption remains influenced by factors such as cost, clinical experience, and healthcare system differences. This review summarizes the current application status, advantages, and challenges of DDS in postoperative chest drainage management after pulmonary resection.

胸腔引流管理是胸外科围手术期管理中的核心环节，其主要作用是排除胸膜腔内积气、血液或其他积液，恢复胸腔负压，促进肺组织复张，维持正常的呼吸力学^[1-3]^。传统水封瓶式胸腔引流系统判断气液量时主要依靠医护人员观察水封瓶气泡溢出、液柱波动和引流液刻度变化，难以做到连续、量化监测，故其主观性十分明显。随着微创手术技术的进步和加速康复外科（enhanced recovery after surgery, ERAS）理念的推广，围手术期管理对客观化、精细化提出了更高要求^[1,4,5]^。近年来发展的数字引流系统（digital drainage system, DDS）能实时、准确地动态监测胸腔内压力变化、积气与积液的引流速率及总量，实现引流数据的数字化、连续性记录与可视化呈现，对胸管管理有很好的指导意义，在胸外科领域逐渐受到关注^[6-8]^。尽管部分研究提示其可改善临床结局，但其应用价值及成本效益仍存在争议。因此，本文对DDS在肺切除术后胸管管理中的应用进展进行综述，并探讨其存在问题及发展方向，旨在为DDS的临床规范应用与后续研究提供循证参考。

## 1 胸腔引流发展历程与DDS的演进

早期胸腔引流主要用于脓胸等严重胸腔感染性疾病的治疗且以开放式引流为主，缺乏密闭防护。随着无菌观念的进步，提出了水封式闭式引流法，奠定了现代胸腔闭式引流的基础^[9]^，并逐步成为治疗胸腔积液、气胸及胸部创伤的标准手段^[10]^。随后进一步发展出三腔引流系统，可同步实现液体收集、气体排出及负压控制，并可联合持续负压吸引装置以优化引流效果^[11]^，该类系统至今仍在临床中广泛应用。伴随ERAS的深入，其局限性日益凸显：（1）对漏气和积液的评估依赖医护人员观察，主观性较强，且难以实现连续、客观的数据记录^[8]^。（2）负压调控精度不足，易受体位、管路阻力及设备稳定性等因素影响，从而影响胸管管理及拔管时机的判断。（3）其对壁式负压装置的依赖在一定程度上限制了患者活动，不利于术后早期康复^[12-16]^。在此背景下，DDS应运而生，实现了从“被动观察”到“主动监测+精准调控”的跨越，为胸腔引流管理提供了新的技术手段。

早期DDS仅能实时定量监测漏气情况，后逐步发展出由管路、集液装置、传感器、监测模块及负压调控系统的一体化DDS。其通过内置传感器可实时监测胸腔压力及气体和液体流量，其中气体流量监测精度可达10 mL/min，液体引流量监测精度可至1 mL，监测数据以数字化形式实时呈现，并可自动连续记录、存储，生成引流趋势曲线，为临床医师提供可量化、客观、连续的漏气和引流液的评估记录^[7,14-18]^。其自带的负压调控系统，可依据预设参数自动调节吸引强度，减少压力波动，将胸膜腔压力维持在预设水平的0.1 cmH_2_O以内，减少了体位、管路等因素导致的负压波动^[8]^。此外，DDS配备多种报警功能，如漏气异常、负压异常和引流阻塞等，提高使用安全性并减轻护理负担^[19]^。基于上述特点，研究^[7,19-22]^表明，DDS有助于提高液气评估的准确性，从而为临床医师判断胸管拔除时机提供更可靠的依据，并可能进一步缩短胸管留置时间及住院时间。

## 2 DDS的优势

### 2.1 积气与积液的客观量化监测

传统胸腔引流系统难以识别短时、间歇性漏气，若判断不当，易造成胸管留置时间延长或拔管过早，进而增加并发症发生风险^[21,23]^。DDS的临床优势已被多项临床研究及meta分析证实。2018年的一项纳入10项随机对照试验的meta分析^[24]^对比了DDS与传统胸腔引流系统的临床结局及成本效益。结果显示，DDS可显著缩短胸管留置时间[加权均数差（weighted mean difference, WMD）为-0.72天，P<0.001]、住院时间（WMD为-0.97天，P<0.001）及漏气持续时间（WMD为-0.95天，P<0.001），同时降低术后医疗费用（减少€443.16，P=0.004），但两组在长时间漏气发生率及患者出院时携带引流设备比例上差异并不显著，提示DDS在积气量化监测的精准性上更具优势，可有效优化拔管决策，且未增加不良结局风险。2022年 Chang 等^[25]^发表的meta分析纳入21项随机对照试验，结果显示在接受肺切除术的患者中，DDS胸管留置时间较吸引式胸腔引流缩短0.68天，住院时间缩短1.4天，其优势得益于DDS对胸腔积气、积液的动态量化监测，可实时反馈引流效果，指导临床调整引流参数。同年， Geraci等^[17]^利用DDS进行术后引流分析，发现与传统胸腔引流系统相比，DDS可检测到更多传统方法易遗漏的短时、间歇性漏气，且这种精准的积气监测与胸管留置时间、住院时间的缩短显著相关。2023年 Zhou等^[20]^检索了12项随机对照试验，对比DDS与传统引流系统在肺切除术后的应用效果，结果显示两组在胸管留置时间[标准化均数差（standardized mean difference, SMD）=-0.49，95%CI: -0.78--0.20）]、住院时长[均数差（mean difference, MD）为-0.79天，95%CI: -1.24--0.34]上均有显著差异，然而，在胸腔引流管夹闭试验次数方面，两组差异无统计学意义（RR=0.74, 95%CI: 0.36-1.49），在持续漏气及心肺并发症发生率方面亦未观察到显著差异，提示DDS主要优势集中于围手术期管理效率优化，而在部分临床干预频率及并发症结局方面仍缺乏一致性证据支持。2024年 Palleiko等^[18]^开展的回顾性研究发现，使用DDS可显著缩短住院时间及胸管留置时间，进一步验证了DDS的临床价值。2025年 Yang等^[26]^也得到了类似的结论，此外，研究还发现，DDS组患者术后前2天睡眠时间更长，下床活动次数更多，推测与DDS精准量化监测减少胸管相关不适、降低重复影像学检查需求有关，间接体现了DDS在积气、积液监测中的优势对患者术后康复的促进作用。

目前若干DDS设备（DigiVent、Thopaz+、Drentech、Thoraguard和ATMOS等）已具备便携性，在维持稳定负压的基础上有利于患者术后早期下床活动，促进肺功能恢复，同时降低深静脉血栓风险，改善患者整体治疗结局^[1,26-28]^，与ERAS理念高度契合，切实改善患者的整体治疗结局^[29,30]^。但不同设备在数据采样频率、漏气检测灵敏度及功能设计方面仍存在差异，这可能也是相关研究结果异质性的重要来源。

### 2.2 为胸管管理标准化提供客观依据

胸管拔除时机是胸腔引流管理中的核心环节，也是临床决策的重点与难点。拔管过早易导致气胸复发、胸腔积液潴留，需二次行胸腔穿刺或置管；拔管过晚则会增加感染风险、延长住院时间、加重患者疼痛^[31]^。当前胸管管理标准化的核心在于缺乏客观、统一、可量化的拔管指标。不同设备之间测量差异、手术方式差异、漏气类型不同、临床风险接受程度不同，对拔管阈值的判断存在极大异质性，难以形成标准化的管理流程^[32]^。DDS通过客观数据消除主观判断差异，为建立统一、规范的胸管管理流程提供循证支撑。

Shintani等^[33]^分析了299例接受肺切除术并使用DDS患者的数据，首次将术后漏气模式分为4类：无漏气（n=217, 73%）、间歇性漏气（n=21, 7%）、漏气量减少（n=40, 13%）和漏气量波动（n=21, 7%）。结果显示，不同漏气分型与患者预后直接相关：无漏气组患者持续性术后漏气（prolonged air leak, PAL）发生率仅0.5%（1/217），而漏气量波动组高达76%（16/21）；间歇性漏气组（24%, 5/21）与漏气量波动组患者的漏气持续时间和胸管留置时间显著延长；与其他组相比，漏气量波动组术后行介入治疗的频率显著更高；间歇性漏气组患者存在拔管后气胸、皮下气肿加重需再次置管的风险。研究证实，DDS可通过连续量化监测实现术后漏气的精准分型，有效预测漏气持续时间和不良事件风险，更重要的是，基于连续量化的评估方式，可减少主观判断差异，为建立术后漏气的标准评估、进行分层管理提供客观依据。基于DDS的漏气量化监测能力，国内外学者逐步探索并验证了标准化的拔管漏气阈值，弥补了传统系统无量化拔管标准的空白。目前多数研究认为，当术后漏气量低于20-50 mL/min且持续稳定6-12 h，可作为安全拔管的重要参考标准^[15,34]^。其中，≤20 mL/min的阈值为保守，≤50 mL/min的阈值在保证拔管安全性的同时，可显著缩短胸管留置时间，更适用于肺叶/肺段切除等微创手术的低中危患者。但需客观认识到，目前不同研究中推荐的拔管阈值仍存在较大异质性，其核心原因在于不同品牌DDS的监测精度、纳入患者的手术方式与风险分层存在差异，尚未形成全球统一的行业标准。

除漏气情况外，24 h胸腔引流量是胸管拔管的另一核心评估指标，但目前尚无基于高级别循证医学证据的统一积液量拔管阈值。临床决策多基于医师的临床经验，而非标准化的循证依据^[35]^。传统上临床多采用24 h引流量200-400 mL作为拔管临界值，而新近指南^[2]^已将安全拔管上限放宽至≤450 mL/24 h。阈值的放宽理论上可缩短胸管留置时间，但也带来了拔管后胸腔积液潴留需再次干预的潜在风险，行业内仍存在较大争议。争议的核心根源在于传统引流系统仅能监测24 h总引流量，无法区分引流液的动态变化趋势。而DDS可实现胸腔引流量的连续监测，实时反映引流液的渗出速率变化，为拔管决策提供更精细化的客观数据，推动引流液拔管阈值从“固定数值界限”向“动态趋势评估”转变。随着信息技术的发展，DDS有望进一步向智能化和远程化方向拓展，并在促进个体化与精准化管理的同时，推动胸腔引流管理流程的标准化与规范化。

### 2.3 精准负压调控与PAL监测

PAL是肺切除术后常见并发症，可导致胸管留置时间延长、住院时间增加，并增加胸腔感染、肺不张等相关风险。胸腔负压管理是促进肺复张及控制术后漏气的重要环节，但目前关于肺切除术后最佳负压策略仍存在争议。传统三腔引流系统主要依赖外部吸引装置维持负压，易受到患者体位变化、管路阻力及设备稳定性等因素影响，导致设定负压与实际作用压力存在偏差^[20]^。负压过高可能增加肺切缘牵拉及组织损伤风险，加重肺泡漏气；而负压不足则可能影响胸腔积气排出及肺复张过程^[21,36]^。DDS通过内置传感器实时监测胸腔压力及漏气变化，并结合主动负压调节功能，使术后胸腔压力管理由固定模式向动态调控模式转变，为个体化胸腔管理提供技术基础^[25]^。

目前，关于DDS负压调控的临床获益尚未形成统一结论。Mitsui等^[21]^研究显示，与中、高负压吸引相比，低负压（5 cmH_2_O）可显著缩短肺切除术后漏气持续时间，提示降低负压水平可能有利于漏气恢复。Pang等^[37]^研究发现，负压水平是影响漏气持续时间的独立因素，高负压组（9-12 cmH_2_O）患者漏气持续时间较低负压组明显延长，进一步支持低负压管理策略的潜在价值。

然而，其他研究并未完全支持低负压优势。Maxwell等^[34]^利用DDS比较标准负压吸引（20 cmH_2_O）与低负压吸引（8 cmH_2_O），结果显示两组在漏气持续时间、胸管留置时间、住院时间及PAL发生率方面均无显著差异，但低负压组术后胸膜介入比例及皮下气肿发生比例有所增加，提示部分患者采用标准负压可能更具安全性。Honda等^[38]^亦发现，与DDS低负压吸引相比，传统水封非吸引管理在缩短漏气持续时间及胸管留置时间方面表现更优，提示术后负压策略可能需要根据患者情况进行选择，而非单纯追求低负压。

上述研究提示，肺切除术后并不存在适用于所有患者的最佳负压模式，负压调控效果可能受到手术方式、肺组织条件、漏气类型及患者基础风险等因素影响。DDS的真正优势并非单纯提供某一固定负压值，而在于能够连续监测胸腔状态变化，为负压调整及胸管管理提供动态依据。研究者开始利用DDS记录漏气速率、胸腔内压力变化等时间序列数据，通过统计学模型及预测算法分析术后漏气模式，预测PAL发生风险，并在术后早期识别高风险患者以优化胸管管理策略，逐步成为未来基于数据驱动的智能化胸腔引流管理的可靠基础^[15,39]^。此外，DDS所产生的连续时间序列数据为术后漏气预测提供了新的研究方向。研究者可通过分析漏气速率变化趋势，建立预测模型，实现对PAL风险及拔管时机的提前判断。Yeung等^[40]^基于DDS采集的术后漏气数据建立时间序列预测模型，结果显示该模型能够预测24 h内漏气闭合趋势，为临床制定拔管策略及评估带管出院的可行性提供了客观依据。

## 3 DDS临床推广中的挑战与局限性

### 3.1 设备操作学习曲线与医护认知适配障碍

尽管DDS在技术上具有诸多优势，但在实际临床推广过程中存在一定局限，具体表现为3个方面：（1）设备操作学习曲线的制约：相较于传统三腔引流系统，其结构更复杂、功能更全面，涵盖参数设置、数据解读、报警处理等多个环节，医护人员在设备引入初期需经过系统培训，医护人员需逐步掌握设备操作及参数解读方法，这一过程可能在短期内影响临床决策模式^[14,20,28]^。（2）诊疗认知重构的困难：从依赖水封瓶气泡观察的经验性判断，到以参数和趋势曲线为主的客观性判断，要求医护人员彻底打破经验依赖，建立“数据驱动”的诊疗认知体系，这种认知转变需长期实践积累，部分医护人员（尤其是工作年限较长、习惯传统模式的人员）接受度较低^[12,14,17]^。（3）护理工作负担的短期增加：DDS的智能报警系统虽能提升临床安全性，但部分护理人员对报警触发机制及异常处理方式尚不熟悉，可能增加初期的工作负担^[6,18,20,41]^。因此，在推广DDS过程中，标准化培训及规范操作是提高医护人员接受度和使用效率的核心路径。随着使用经验的积累，医护人员对DDS的接受度逐渐提高，有助于增强临床决策信心及护理工作效率。

### 3.2 国外获益明显而国内证据有限的成本效益差异

需要关注的是，DDS在不同国家及医疗体系中的临床效益和成本效益存在一定差异。目前学界对此主要存在两种观点：（1）有研究^[8]^认为DDS通过客观量化积气、积液及漏气情况，可优化拔管决策，缩短胸管留置时间及住院时间，减少术后并发症相关的医疗支出，进而在整体上降低医疗费用；（2）也有研究^[42]^指出，在医护资源充足或床位周转压力较小的医疗环境中，DDS缩短住院时间所带来的经济学优势未必能够充分体现，其较高的初始投入反而会增加医疗成本。

国外研究总体提示DDS在缩短胸管留置时间及住院时间的同时，具有一定成本节约潜力。Brunelli等^[43]^的随机对照试验显示，与传统引流系统相比，DDS平均节约总体医疗费用€476。Jablonski等^[44]^研究亦报道类似结果，提示费用减少€400-500。英国国家健康与护理卓越研究所的卫生经济学评估^[45]^亦指出，DDS可通过减少引流时间及住院时间降低医疗支出，估算每例患者可节省约£111.33，并对其临床应用持积极推荐态度。总体而言，国外证据较一致支持DDS在特定医疗体系中具有一定成本效益，其主要来源于住院周期缩短及资源消耗减少。相比之下，国内研究尚未一致证实DDS在成本控制方面的明确优势。多项单中心回顾性研究^[46-48]^提示DDS与传统引流方式在总住院费用方面差异无统计学意义，部分研究虽观察到费用下降趋势，但证据质量有限且结论不一致^[49]^。其原因可能与我国ERAS理念广泛应用后住院时间显著缩短有关，使DDS进一步缩短住院时间的效益有限。从经济学角度来看，DDS的初始采购成本、维护成本及人员培训成本通常高于传统胸腔引流系统，这一成本差距成为其在医疗资源有限的地区推广应用的重要阻碍^[24,41]^。因此，DDS的成本效益受医疗体系与资源配置背景影响较大。未来仍需基于我国真实医疗环境开展多中心前瞻性研究，并结合卫生经济学方法进行系统评估。

## 4 展望

现有证据总体提示，DDS在缩短胸管留置时间、住院时间及漏气持续时间等方面具有一定优势，但其对并发症、持续漏气及成本效益等结局的影响仍存在一定异质性。不同设备在监测能力、功能设计及临床应用特点方面存在一定差异。Thopaz+主要通过连续定量监测空气流量，并结合漏气趋势分析功能，为临床医师评估漏气恢复过程及判断拔管时机提供参考；同时具备液体监测、负压调节及数据记录功能。DigiVent侧重于空气流量监测及胸腔压力信息采集，其压力监测模块有助于评估术后胸腔状态变化。Drentech Digital可实现空气流量及引流量监测，并通过综合引流管理功能促进胸管管理流程规范化。Thoraguard则结合气流与压力监测功能，并配备报警及压力控制模块，有助于异常情况识别及安全管理。ATMOS主要提供气流、负压及引流量监测，并采用便携化设计，可能有利于促进患者术后早期活动。

总体而言，不同DDS设备均具备连续量化监测和数据化管理的核心特点，但在监测参数范围、压力控制方式、报警功能及数据管理模块等方面存在差异。目前多数临床研究主要将DDS作为整体技术进行评价，尚缺乏不同设备之间的直接比较，因此上述功能差异是否能够进一步转化为临床结局优势仍需高质量研究验证。

此外，目前仍缺乏基于DDS数据的标准化个体化管理方案。已有研究^[8,50-52]^表明，手术方式及患者基础因素与PAL发生风险密切相关，而DDS能够提供连续动态监测数据，为风险分层管理提供技术基础。针对不同手术类型（肺叶切除、肺段切除、全肺切除）及高风险人群（高龄、基础肺功能差等）的DDS应用策略，目前仅为推测性分析，临床价值有待进一步明确。

近年来，DDS连续采集的漏气及压力变化数据为预测模型建立提供了新的可能。研究者尝试利用DDS时间序列数据预测术后漏气恢复趋势，为拔管决策及带管出院评估提供参考。未来结合人工智能及机器学习技术，有望进一步提高DDS数据利用价值，实现术后风险预测和个体化管理。

除肺切除术后胸管管理外，DDS在其他胸外科疾病中的应用价值也值得进一步探索，如通过动态监测漏气趋势评估支气管胸膜瘘患者恢复情况，以及通过引流液变化评价脓胸治疗效果。此外，随着设备便携化的发展，DDS可能为部分患者带管出院及远程随访提供技术支持，但相关应用仍需更多临床研究验证。

目前DDS相关证据仍存在一定局限性。现有研究受样本量、研究设计及围手术期管理方案差异的影响，存在一定异质性；不同研究在拔管标准、漏气定义及患者选择方面尚未完全统一，限制了研究结果的进一步推广。未来仍需开展多中心、高质量随机对照研究，并结合卫生经济学评价，进一步明确DDS的最佳应用人群及临床价值。

## 5 总结

DDS的核心价值在于让胸管管理从经验判断逐步转向基于数据的客观决策，通过连续、客观地监测胸腔压力及气液流量，实现精确量化评估，为胸管拔除时机和术后管理提供可靠依据；然而，其临床应用仍受医疗体系、成本及医护人员操作经验影响，其优势在较短的住院时间中可能并不明显。随着ERAS理念的推广、设备便携化及网络传输，有望实现带管出院及远程动态监测，逐步向个体化、精细化和远程化迈进。未来结合多中心前瞻性研究与智能化分析，数据驱动的术后管理优化及预测并发症风险更加可行，DDS正逐步成为胸外科围手术期管理的重要工具。
